# Retraction Note: Purification and Characterization of Desferrioxamine B of Pseudomonas fluorescens and Its Application to Improve Oil Content, Nutrient Uptake, and Plant Growth in Peanuts

**DOI:** 10.1007/s00248-026-02838-8

**Published:** 2026-07-25

**Authors:** S. Nithyapriya, Lalitha Sundaram, Sakthi Uma Devi Eswaran, Kahkashan Perveen, Najla A. Alshaikh, R. Z. Sayyed, Andrea Mastinu

**Affiliations:** 1 PG and Research Department of Botany, Padmavani Arts and Science College for Women, Salem, 636011 India; 2 Department of Botany, Periyar University, Salem, 636011 India; 3 Department of Botany and Microbiology, College of Science, King Saud University, PO Box -2455, Riyadh, 11451 Saudi Arabia; 4 Department of Microbiology, G B Patel Science, PSGVP Mandal’s S I Patil Arts, STKV Sangh Commerce College, Shahada, 425409 India; 5 Faculty of Health and Life Sciences, INTI International University, Negeri Sembilan, Persiaran Perdana BBN, Putra Nilai, 71800 Nilai, Malaysia; 6 Department of Molecular and Translational Medicine, Division of Pharmacology, University of Brescia, Brescia, 25123 Italy

**Retraction Note: Microbial Ecology (2024) 87:60**

10.1007/s00248-024-02377-0

RETRACTION NOTE.

The Editor-in-Chief has retracted this article. Following the publication, multiple concerns have been raised regarding incorrect statements and unsupported conclusions (See below). The authors were contacted to provide an explanation of concerns, however, they were unable to provide a satisfactory explanation.

• The structure in Fig. 7 is labeled as desferrioxamine B which is incorrect. It is the structure of ferrichrome.

• The identification of the siderophore as desferrioxamine B is not supported by the data shown.

• The statement ‘Centrifuging a product at 1000 rpm’ is incorrect.

• C-13 data present in Fig. 6 does not show a list of assignments.

• The study hints at a positive effect of the bacterium on plant growth but the results are not compelling.

The Editor-in-Chief therefore has lost confidence in the content of this article.

S. Nithyapriya, Lalitha Sundaram, Sakthi Uma Devi Eswaran, Kahkashan Perveen, R.Z. Sayyed, and Andrea Mastinu disagree with this retraction. Najla A. Alshaikh did not respond to correspondence from the Publisher regarding this retraction.

